# The Associations between Lifestyle Habits and Anxiety: A Prospective Study on Adolescents One Year after the Outbreak of the COVID-19 Pandemic

**DOI:** 10.3390/children11030282

**Published:** 2024-02-25

**Authors:** Laura Pedrini, Serena Meloni, Julia Dawson, Andrea Geviti, Natale Salvatore Bonfiglio, Anna Cattaneo, Roberta Rossi

**Affiliations:** 1 Unit of Psychiatry, IRCCS Istituto Centro San Giovanni di Dio Fatebenefratelli, Via Pilastroni, 4, 25125 Brescia, Italy; smeloni@fatebenefratelli.eu (S.M.); jdawson@fatebenefratelli.eu (J.D.); rrossi@fatebenefratelli.eu (R.R.); 2 Statistics Service, IRCCS Istituto Centro San Giovanni di Dio Fatebenefratelli, Via Pilastroni, 4, 25125 Brescia, Italy; ageviti@fatebenefratelli.eu (A.G.); nbonfiglio@fatebenefratelli.eu (N.S.B.); 3 Biological Psychiatry Unit, IRCCS Istituto Centro San Giovanni di Dio Fatebenefratelli, Via Pilastroni, 4, 25125 Brescia, Italy; acattaneo@fatebenefratelli.eu; 4 Department of Pharmacological and Biomolecular Sciences, University of Milan, 20133 Milan, Italy

**Keywords:** adolescents, lifestyle, sleep, internet use, screen use

## Abstract

Changes in lifestyle during the pandemic may have predisposed adolescents to vulnerability to poor mental health. This study aims to evaluate these changes and their association with the course of anxiety. A prospective study was conducted with 153 participants (16 years old, 72% female) who were assessed before the pandemic (T0, November 2019–January 2020) and one year later (T1, April–May 2021). Lifestyle habits (free-time activities, maladaptive behaviors, sleep, screen use) and anxiety were measured. Data concerning experiences related to COVID-19 and family relations during lockdown were collected. A worsening in lifestyle habits and anxiety was found. Of note, the pattern of associations between lifestyle habits and anxiety was quite different in the two time-points, suggesting that the purpose and the impact of some habits may be changed after the pandemic. Regression analyses showed that increases in anxiety were associated with increases in sleep problems, heightened efforts to reduce screen time, and loneliness. Pathway analysis revealed the absence of cross-lagged effects among anxiety, screen use, and sleep, while concurrent associations between variables were found in both the assessments. These results suggest possible long-term effects of the pandemic. Risk-factors associated with the course of anxiety were identified among lifestyle habits, thus contributing to identifying targets for interventions.

## 1. Introduction

The restrictions that have been formulated to contain the COVID-19 pandemic, such as social distancing, school closures, and halting sports activities, directly influenced adolescents’ daily routines. Several studies that were conducted during the first phase of the pandemic reported changes in the lifestyles of adolescents such as worsened sleep quality [1,2,3,4,5], increased amount of time spent online [6,7,8], and modifications in dietary habits [9].

Overall, these changes in habits may have predisposed adolescents to a higher vulnerability to poor mental health because they may have affected relevant developmental milestones [10]. For instance, it is well known that relationships with peers play a crucial role in the process associated with identity formation; therefore, if these relationships are disrupted, it may lead to loneliness and emotional problems [11,12]. Moreover, a large body of research shows that sleep efficiency promotes emotion regulation, alertness, and impulse control, whereas sleep-related problems have been found to be associated with a decrease in positive affect and with an increase in anxiety [13]. Finally, the digital world offers a spectrum of benefits and drawbacks: on one hand, it serves as a crucial avenue for adolescents to connect with peers and momentarily divert their attention from challenges. On the other hand, certain individuals might encounter difficulties in effectively self-regulating their engagement with digital technologies, potentially exacerbating pre-existing mental health problems [14,15,16].

Epidemiological studies conducted during the pandemic reveal a rising trend in clinically heightened anxiety symptoms over time among adolescents, aligning with concerns about COVID-19′s effects on this demographic [6,17,18]. At the same time, it has been pointed out that not all young people showed a deterioration in their psychological health during the pandemic [19,20,21]. Therefore, long-term, accurate monitoring to identify risk and resilience factors associated with different mental health paths has gained importance. In this framework, it could be useful to evaluate the association between adolescents’ lifestyle habits and mental health conditions. However, most of the studies on this topic are cross-sectional, with only a limited number offering evidence clarifying the mechanisms behind the associations with mental health.

Based on this knowledge, we conducted a prospective study with the following aims: (i) to compare lifestyle habits (i.e., free-time activities, maladaptive behaviors, sleep quality, screen use) reported by students just before the pandemic and one year later. We hypothesized a trend of change in lifestyle habits as shown by previous studies conducted during the first lockdown; (ii) to evaluate the association across time between anxiety, lifestyle habits, and the variables related to the experience of the lockdown. We hypothesized that some habits became more frequent among adolescents and may not be distinguishable from those of adolescents with different levels of anxiety. Moreover, we hypothesized that changes in some behaviors are associated with changes in anxiety, thus helping to explain the different reactions to stress induced by the pandemic; (iii) to deepen the investigation concerning the relations between the course of sleep problems over time, screen use, and anxiety. Specifically, we tested the same model with both cross-lagged effects and concurrent associations among these variables, thus contributing to clarifying the association in such relevant domains.

## 2. Materials and Methods

This is a prospective study conducted as part of a randomized clinical trial (RCT) on emotional dysregulation in adolescents (ClinicalTrials.gov ID: NCT04349709). The study was proposed to a high school in a city located in Northern Italy. Each school independently selected at least two classes in the 10th grade to participate. Following the protocol of the initial study, students with certified diagnoses of autism spectrum disorders were excluded.

For the purpose of the present study, we included a sample assessed at two time-points: a pre-COVID baseline (T0) with data collection from November 2019 to January 2020 and a 1-year follow-up assessment (T1) with data collection during April and May 2021. Specifically, the sample included 153 students from eight classes in three high-schools.

Both the assessments were arranged during regular school day hours: at T0 participants completed a paper-based battery of questionnaires at school, while at T1 they completed an online version of the same battery. The researchers were available for students during both the sessions.

This study was approved by the local ethics committee (approval n.40/2019 on 11 July 2019 and n.13/2021 on 26 February 2021) and was carried out following the ethical standards presented in the 1964 Declaration of Helsinki. Written informed consent was obtained both at T0 and T1 from all subjects.

The dataset of the current study is available in the Zenodo repository (Zenodo DOI 10.5281/zenodo.10090891).

### 2.1. Assessment

The following variables were assessed both at baseline (T0) and one year later (T1):

*Free-time activities*. Participants were asked to describe their free-time activities by reporting the amount of daily time (1) spent with friends, (2) watching TV, (3) reading, (4) and being inactive and (5) the amount of weekly time spent playing sports on a four-level scale (1 = not at all, 2 = up to 1 h, 3 = from 1 to 2 h, 4 = more than 2 h).

*Maladaptive behaviors*. Participants were asked to report the frequency of the following maladaptive behaviors: (a) binge eating, (b) binge drinking behaviors, and (c) cannabis use in three separate questions. Subjects could respond based on the following frequency scale: 0 = never; 1 = only one time; 2 = once a week; 3 = two or three times a week; 4 = at least 4 times a week.

*Sleep quality*. Participants were asked to describe their sleep quality by answering the following questions [22]: (1) Well rested = “How many of the past seven days did you get enough sleep so that you felt rested when you woke up in the morning?” (response options: from 0 to 7 days); (2) Early awakening = “In the past seven days, how often have you awakened too early in the morning and couldn’t get back to sleep?” (response options: from 0 to 7 days); (3) Difficulties falling asleep = “In the past seven days, how often have you had an extremely hard time falling asleep?” (response options: from 0 to 7 days); (4) Problems with daytime sleepiness = “In the past seven days, how much of a problem have you had with sleepiness during your daytime activities?” (response options: 1 = no problem at all; 2 = a little problem; 3 = more than a little problem; 4 = a big problem; 5 = a very big problem).

*Screen use*. Participants were asked to describe their use of social media or the internet during last month by answering the following four questions: (1) How many hours do you usually use social media/internet per day? (0 = up to one hour; 1 = from 1 to 2 h; 2 = from 3 to 5 h; 3 = more than 5 h). (2) How frequently do you find yourself staying up late to spend more time online/on the internet? (3) How often have you found yourself having little time to study because of the time spent online/on the internet? (4) How often have you found yourself reducing the amount of time you spend online/on the internet? Response options for questions 2, 3, 4 were as follows: 0 = never, 1 = only once; 2 = once a week; 3 = at least twice a week. In order to better describe problems related to screen use we calculated the “Problematic screen use” variable. To this purpose, we considered both the amount of daily screen time and related problems. Specifically, participants spending more than 2 h online daily [23] and scoring “3” on at least one of questions 2, 3, or 4 were classified as having “problematic screen use”.

*Anxiety*. The Screen for Child Anxiety-Related Emotional Disorders (SCARED) [24] was used to measure anxiety. The SCARED is a 38-item self-report questionnaire measuring anxiety with the following five subscales: panic disorder, generalized anxiety disorder, separation anxiety disorder, school anxiety, and social anxiety. Each item is rated on three-point scale (1 = almost never, 2 = sometimes, 3 = often). The sum of all the items results in the total score. The Italian version of the SCARED showed good internal consistency (Cronbach’s alpha ranged from 0.66 to 0.94) [25].

Moreover, at T1 we also registered the following data:

*COVID-19-related experience*. Participants were asked to report whether they contracted COVID-19 (yes; no); whether anyone of their family members contracted COVID-19 (yes; no); whether anyone of their family members had been hospitalized due to COVID-19 (yes; no); and whether anyone of their family members died from COVID-19 (yes; no). Moreover, they rated how worried they were about contracting COVID-19 on a 0–10 visual analog scale.

*Lockdown-related experience*. Participants were asked to report whether during lockdown their parents stayed at home (yes; no); whether conflicts in the family increased (yes; no); and whether they felt alone (yes; no).

### 2.2. Data Analysis

Data was cleaned, coded, and evaluated using IBM SPSS Statistics, version 21 [26]. The Jamovi package was used only for the path analysis [27]. The reported confidence intervals were set at 95%. The assumptions of normality and multivariate normality were assessed by visual inspection of the QQ plots and density histograms for each variable. Durbin–Watson tests were used to detect autocorrelation of residuals, variation inflation factors (VIFs) and tolerance indexes were used for collinearity, and Levene’s test was used for homoscedasticity. No significant violations of the assumption were found. The absolute and relative frequencies and the means and standard deviations were calculated for each of the sociodemographic and response variables. The Chi-square test was carried out for categorical variables in order to investigate differences between subgroups at baseline.

In order to achieve a global index of social media/internet use, a new variable called “screen use composite score” was created. It corresponds to the factor score obtained through the principal component analysis (PCA) considering the responses to questions n1, n2, and n3 on the screen use. Only one dimension was extracted (60.2% of explained variance for T0 and 64.3% for T1) with a Cronbach’s alpha of 0.67 for T0 and 0.72 for T1.

In order to evaluate the difference between T0 and T1 for each variable of interest, a series of paired Student *t*-tests, for continuous variables, and McNemar tests, for categorical variables, were calculated.

In order to evaluate the change over time, a series of delta variables was created for each of the variables of interest (i.e., delta = variable at T1—variable at T0). Pearson’s r was used to find correlation between the delta variables. A series of multivariate regression models were then created for those variables that were significantly correlated with the delta of anxiety’s total score.

Finally, a path analysis was performed taking into account the third aim of the study, which was to determine the association between sleep, social media/internet use, and anxiety. Specifically, the following variables assessed at both T0 and T1 were used: (a) total anxiety score, (b) difficulty falling asleep, and (c) online composite score. For model performance, the following fit indexes were used: the Chi-square, the root mean square error approximation (RMSE), the standardized root mean square residual (SRMR), the comparative fit index (CFI), the Tucker–Lewis index (TLI), and the goodness of fit (GFI) [28,29].

## 3. Results

Participants were mainly female (72%), and the average age was 16 years old (16.1 ± 0.49). A total of 70 students (46%) reported that at least one of their family members contracted COVID-19. A total of 24 (16%) students reported that at least one of their family members had been hospitalized for COVID-19 treatment, and n = 15 (10%) students reported that at least one of their family members died of COVID-19. A total of 19 (12%) students contracted COVID-19. On average, participants expressed moderate levels of concern about the possibility of contracting the virus on a 0–10 visual analog scale (5.5 ± 2.1).

### 3.1. Lifestyle Habits and Anxiety across Time

As reported in Table 1, there was a decrease in the amount of time spent with friends (*p* < 0.001) and in reading (*p* < 0.001). The frequency of binge-eating episodes was higher at follow-up compared to the pre-pandemic period (*p* = 0.003). Moreover, there was an overall increase in difficulties falling asleep (*p* = 0.036) and daytime sleepiness (*p* = 0.011). The time spent online increased over time (*p* < 0.001). In particular, those that declared spending more than 2 h a day online were n = 94 (61%) and n = 122 (80%), respectively, at baseline and at follow-up (without considering online learning). After the pandemic, a relevant number of students (n = 43, 28.1%) stated that they spent more than 5 h online a day (without considering online learning). There was an increase in the proportion of adolescents frequently staying up late to spend more time online (*p* < 0.001), as well as trying to reduce the amount of time spent online (*p* < 0.001). Finally, the proportion of students classified as having “Problematic screen use” significantly increased over time (n = 59; 38.6% at T0; n = 93, 60.8% at T1, *p* = < 0.001).

Anxiety levels increased across time, as shown by the difference in the total SCARED mean score (64.9 ± 12.1 at T0 and 67 ± 12.6 at T1; *p* = 0.002) and in the following subscales: generalized anxiety (19.4 ± 3.9 at T0 and 20.4 ± 4.2 at T1; *p* < 0.001) and school anxiety (6.8 ± 1.9 at T0 and 7.6 ± 2.3 at T1; *p* < 0.001). In contrast, we did not find relevant differences for panic symptoms (20.2 ± 5.8 at T0 and 20.4 ± 5.6 at T1; *p* = 0.383), separation anxiety (11.2 ± 2.5 at T0 and 11.1 ± 2.6 at T1; *p* = 0.574), and social anxiety (7.6 ± 2.4 at T0 and 7.7 ± 2.6 at T1; *p* = 0.400).

### 3.2. Associations between Anxiety and Current Lifestyle Habits

Table 2 shows the associations between anxiety and lifestyle habits that were measured at the same time. Findings show that, before the COVID-19 pandemic, anxiety was associated with time spent with friends, sports practice, binge-eating episodes, and sleep-related problems. These associations were also confirmed at the 1-year follow up. Moreover, at T0, higher levels of anxiety were associated with the number of hours spent inactive and the number of hours spent online, whereas our findings at the 1-year follow up no longer indicated an association between these behaviors and levels of anxiety. An inverse correlation between anxiety and the feeling of being well-rested upon waking up emerged after the pandemic. Finally, after the pandemic higher anxiety levels were significantly associated with dysfunctional internet use (i.e., staying up late to spend more time online, reduced time to study, ineffective attempts to reduce screen use), rather than with the number of hours spent online as found at baseline.

### 3.3. Factors Associated with the Change in Anxiety Levels over Time

In order to test possible predictors of longitudinal change in anxiety symptoms, separated models of regression analysis were prepared. We considered as potential predictors both the changes in lifestyle habits (delta variables) and the factors related to the experience of the first lockdown. Specifically, among lifestyle habits we considered only the variables significantly correlated with changes in anxiety, which were the variations in difficulty in falling asleep (r = 0.32; *p* < 0.001) and in attempts to reduce screen time use (r = 0.24; *p* = 0.002).

Our findings show that the increase in difficulties in falling asleep is associated with the increase in anxiety levels over time (*p* < 0.001). Moreover, participants reporting an increase in ineffective attempts to reduce the amount of time spent online were more likely to record an increase in anxiety (*p* = 0.003). Concerning the variables related to the experience of the first lockdown, we found no evidence for an effect of home climate (i.e., parents at home, increased family conflicts) on levels of anxiety. In contrast, having experienced loneliness during the first lockdown (*p* = 0.027) and having experienced the death of a family member (*p* = 0.012) were associated with increased anxiety levels over time. Subsequently, the statistically significant variables were simultaneously entered in the same model (Table 3). All of them, except for the one related to having experienced death of a family member due to COVID-19, continued to be significantly associated with the course of anxiety symptoms. Specifically, it was found that the increase in difficulties in falling asleep (*p* < 0.001; standardized *β* = +0.27) and in the attempts to reduce the amount of time spent online (*p* = 0.005; standardized *β* = +0.21) and having experienced feelings of loneliness during lockdown (*p* = 0.011; standardized *β* = −0.19) were predictors of worsened anxiety symptoms over time, even if the standardized coefficients suggest small effects.

### 3.4. Cross-Lagged and Concurrent Associations among Sleep, Screen Use, and Anxiety

**Figure 1:**
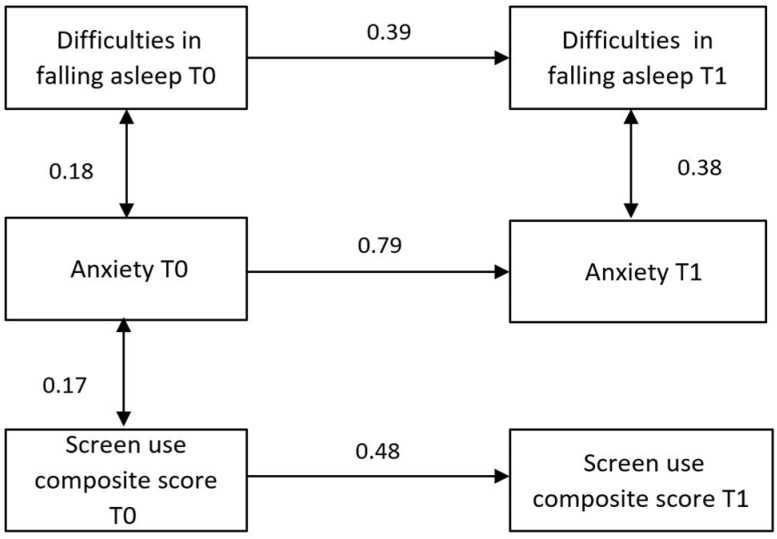
Path model with beta coefficients. Beta values are shown for each unidirectional and bidirectional interaction in arrows. Normality and Mardia tests revealed only skewness and not kurtosis tendencies (Skewness: 2 = 85; df = 56; *p* = 0.007; Kurtosis: z = 0.81; *p* = 0.419); hence, the robust maximum likelihood technique was employed to estimate SEM parameters. Several fit indexes revealed the goodness of the model: the Chi-square value was 11.5 (df = 9; *p* = 0.244); the RMSEA was 0.042; the standardized version (SRMSE) was 0.055; the Tucker–Lewis Index (TLI) was 0.990; the comparative fit index (CFI) was 0.983; and the GFI was 0.998.

Based on the significant associations found among sleep problems, screen use, and anxiety, a pathway analysis was conducted in order to explore their relationships at the two time-points. Specifically, we tested both concurrent association and cross-lagged effects. As shown in Figure 1, the results revealed no significant cross-lagged associations, meaning that none of the variables measured at T0 was associated with the others measured at T1 (for example: anxiety at T0 was not associated with screen use, nor with sleep at T1). Concurrent associations were found between anxiety and sleep in both the assessments; that is, anxiety and sleep were associated at both time points of the assessment. while screen use was associated with anxiety only before the pandemic. According to recognized standards, the model appears to be very acceptable based on the fit index.

## 4. Discussion

The present data refers to a period with less stringent restrictions, as it was one year after the outbreak of COVID-19; therefore they seem to suggest a possible long-term effect of the pandemic on adolescents’ daily routines. Indeed, similarly to the first wave of the pandemic [1,2,3,4,5,6,7,8], we found a decrease in time spent with friends and reading, whereas sleep-related problems, binge-eating episodes, and screen use increased over time. Of note, staying up too late to spend more time online was the behavior that showed the strongest effect size, thus suggesting that sleep quality may have been particularly affected during this period.

Interestingly, the variables associated with anxiety were slightly different in the two time-points, suggesting that certain behaviors may have taken on different purposes and consequences in daily routines. Before the pandemic, anxiety showed an inverse correlation with time spent with friends and engagement in sports activities. Conversely, it was directly associated with time spent inactive, binge-eating episodes, sleep-related problems, and online time. These findings align with the well-established literature on the relationship between lifestyle and mental health [11,12,13,14] and emphasize the importance of interventions aimed at promoting functional habits to support mental health. After the pandemic, some behaviors (i.e., time spent inactive and the amount of time spent online) were no longer significantly associated with anxiety. It is important to note that during the lockdown period, increases in inactivity and heightened screen use became common behaviors among adolescents. As a result, it seems that these behaviors no longer serve as distinguishing factors for the emotional state of youths, as they did prior to the pandemic. In particular, social media may have represented a vital source for fostering peer connections and providing valuable support, especially in the midst of lockdown restrictions. Of note, a study conducted during the pandemic showed that screen time dedicated to fostering connections was associated with reduced feelings of loneliness and heightened levels of overall well-being [9].

Gaining insight into the mechanisms that underscore the connection between lifestyle habits and anxiety holds significance, as it facilitates the identification of potential intervention targets [30]. In our study, the increase in anxiety over time was predicted by having experienced feelings of loneliness during lockdown, loss of a family member due to COVID-19, difficulties in falling asleep, and difficulties in curtailing screen use. These results lead us to hypothesize that these variables may have served as pandemic-induced risk factors, potentially contributing to emotional challenges. The school closure and the resultant inactivity due to the restrictions have undeniably led to alterations in sleep patterns, which may have in turn negatively impacted levels of anxiety. Conversely, there is a plausible scenario wherein the overarching uncertainty stemming from the pandemic could induce a pervasive sense of generalized anxiety or heightened alertness, consequently leading to disruptions in sleep patterns. It is well recognized that sleep-related symptoms and anxiety share an intrinsic connection, and often they overlap [31]. To our knowledge, the only study aiming to investigate the direction of the relationship between sleep and anxiety during the pandemic found support of a bidirectional association between these variables [1]. Interestingly, the results of our pathway analysis showed similar evidence. Based on these findings, interventions designed to cultivate awareness regarding sleep patterns and enhance strategies for upholding a functional daily routine could be useful. In reinforcement of this proposal, we observed that feeling well-rested upon morning awakening exhibited a significant association with reduced anxiety levels exclusively in the second assessment. This highlights the noteworthy influence of good sleep during challenging circumstances such as the pandemic, as it potentially mitigates the adverse impact of stressful events. Indeed, there is evidence documenting the influence of the quality of sleep on effective emotion regulation and positive affect [13].

Another main finding of the present study refers to problematic screen use that emerges as a factor associated with anxiety; this is interesting since the influence of screen use on mental health remains a topic of contention. Increased use of screen technology has been found to be associated with heightened mental health problems [32]. Conversely, a recent review encompassing longitudinal studies reached the conclusion that there exists only a minor direct impact of screen use on psychopathological symptoms [33]. Indeed, according to the “displacement hypothesis”, adverse consequences arise when screen use substitutes other healthy behaviors, such as social interaction, functional problem-solving, and functional lifestyle habits [8,14,16]. Consistent with this theory, after the pandemic our sample yielded no correlation between anxiety and the number of hours spent online, whereas we observed an association between anxiety and the presence of dysfunctional screen use (i.e., staying up late to spend more time online, time to study negatively affected by the amount of time spent online, ineffective attempts to reduce amount of time spent online). We can speculate that challenges in effectively regulating screen use, rather than the mere duration of time spent online, might play a role in exacerbating anxiety. In line with this interpretation, a review of the studies conducted on clinical samples of youth patients has demonstrated that certain young individuals might encounter challenges in self-regulating their screen-time, which in turn could amplify psychological distress [15]. Similarly, another study showed that the impact of screen use on mental health may be mediated by coping strategies. Adolescents who tend to adopt functional coping strategies appear to exhibit greater resilience against the depressive symptoms associated with screen time [16]. Considering this evidence, the findings of the present study are valuable in identifying a risk factor for poor mental health in adolescents during the pandemic. The study highlights a potential unfavorable condition for a significant portion of the sample, as we observed an increase from 39% to 61% of participants with problematic screen use.

Lastly, loneliness emerges as another pivotal factor for higher levels of anxiety, and this finding is in line with several studies conducted on young people during the COVID-19 pandemic [34]. According to the cognitive model of anxiety [35], perceiving a lack of support intensifies feelings of threat amid stress-inducing circumstances, and this may explain the association between feelings of loneliness and anxiety. Loneliness is an unpleasant and distressing condition that results from deficiencies in an individual’s social connections, but it is also a subjective experience that refers to cognitive processes [36]. In this regard, in a large sample of adolescents it was found that the association between loneliness and psychopathological symptoms during lockdown was independent from social contacts, whereas it was significantly associated with rejection sensitivity (i.e., the inclination to anticipate social rejection with anxiety, promptly perceive it, and react excessively) [11]. The results of our study contribute to the existing literature by showing the importance of cognitive processes; indeed, we found that higher levels of anxiety were associated with feelings of loneliness during lockdown, whereas the variables closely tied to social interactions at home (i.e., having parents at home and family conflicts at home during lockdown) did not exhibit any significant association with anxiety. Of course, we cannot exclude that the prohibition of meeting friends, rather than the quality of family relationships, may have contributed to the experience of loneliness. However, it is also possible that our data underscores the importance of intrapersonal variables in mediating the impact of loneliness, leading to hypotheses of intervention. Specifically, since negative interpretation bias may serve as a mechanism through which loneliness becomes a precursor to emotional difficulties, interventions aimed at challenging these negative interpretations could be useful in mitigating mental health symptoms [37].

### Strengths and Limitations

The present study has several strengths, with its prospective design and one-year follow-up being foremost among them. Additionally, the response rate enhances the reliability of the findings. The sample was sourced from a homogeneous geographical area which experienced a significant impact from COVID-19. The assessment of internet use in our study provides more comprehensive information compared to existing studies conducted during pandemic. While many previous studies only considered the number of hours spent online, our assessment also evaluated the impact on personal functioning. Finally, while the analyses offer valuable insights into the potential influence of the pandemic on the course of adolescents’ anxiety, it is crucial to interpret these findings within the framework of the study’s limitations. Indeed, it is important to acknowledge that the convenience-based sampling approach we employed, along with the high proportion of female participants, may limit the generalizability of our findings to the broader population of adolescents. Moreover, the lack of a sample size calculation to assess the power of the analysis and the small effects observed for some of the factors predicting the course of anxiety should be taken into consideration. Additionally, while loneliness emerged as a significant factor influencing mental health, it is worth noting that it was assessed using a single item. Lastly, it is important to emphasize that further studies are necessary to comprehensively capture the impact of the pandemic on adolescents’ wellbeing, including aspects such as coping styles and emotional regulation strategies.

## 5. Conclusions

This study is among the limited number of prospective investigations featuring a 1-year follow-up. It sheds light on the increase in anxiety levels and the enduring prevalence of negative lifestyle habits, even during a period characterized by less stringent restrictions. The lifestyle habits associated with anxiety were slightly different at the two assessment points; therefore, future studies should consider that certain behaviors may have taken on different purposes and consequences in daily life. Specifically, after the pandemic, some behaviors (i.e., time spent inactive and the amount of time spent online) were no longer associated with anxiety, while quality of sleep became more strictly associated with anxiety. Moreover, at follow-up, dysfunctional internet use, rather than the sheer number of hours spent online, exhibited an association with anxiety levels. Potential risk factors that might have influenced the course of anxiety were identified, specifically, the increase in problematic screen use, sleep-related problems, and feelings of loneliness during lockdown. Overall, these data support the importance of interventions targeting these domains to improve adolescents’ mental health.

## Figures and Tables

**Table 1:** Lifestyle habits before the pandemic (T0) and one year later (T1).

	T0 N = 153	T1 N = 153	Test (DF)	*p*-Value ^1^	Effect Size $
***Free-time activities***					
Time spent with friends (1–4)	2.93 ± 0.96	2.42 ± 1.01	t = 9.64 (150)	*p* < 0.001	0.40
Time spent watching TV (1–4)	2.03 ± 0.90	1.94 ± 0.97	t = 1.25 (151)	*p* = 0.214	0.10
Time spent reading (1–4)	1.88 ± 0.87	1.69 ± 0.88	t = 3.1 (151)	*p* = 0.002	0.25
Time spent inactive (1–4)	2.31 ± 1.01	2.21 ± 1.05	t = 1.21 (150)	*p* = 0.227	0.10
Time spent playing sports (0–4)	2.16 ± 1.63	1.96 ± 1.55	t = 1.41 (152)	*p* = 0.161	0.11
***Maladaptive behaviors***					
Binge drinking (0–4)	0.18 ± 0.47	0.16 ± 0.49	t = 0.52 (152)	*p* = 0.607	0.04
Binge eating (0–4)	0.12 ± 0.43	0.37 ± 0.97	t = −3 (152)	*p* = 0.003	0.24
Cannabis use (0–4)	0.11 ± 0.47	0.22 ± 0.67	t = −1.76 (152)	*p* = 0.081	0.14
***Sleep quality***					
Enough sleep (0–7)	2.03 ± 1.9	1.77 ± 1.6	t = 1.61 (148)	*p* = 0.110	0.13
Early awakening (0–7)	1.14 ± 1.9	1.03 ± 1.6	t = 0.64 (152)	*p* = 0.524	0.05
Difficulties in falling asleep (0–7)	1.86 ± 2.2	2.30 ± 2.4	t = −2.11 (158)	*p* = 0.036	0.17
Daytime sleepiness (0–4)	1.05 ± 0.93	1.27 ± 1.02	t = −2.58 (152)	*p* = 0.011	0.21
***Screen use***					
Time spent online (1–4)	2.63 ± 0.71	3.05 ± 0.75	t = −6.34 (152)	*p* < 0.001	0.51
Staying up too late to spend more time online (0–5)	1.37 ± 1.42	1.93 ± 1.35	t = −4.44 (152)	*p* < 0.001	1.21
Little time to study because of time spent online (0–5)	1.30 ± 1.32	1.71 ± 1.35	t = −3.39 (152)	*p* < 0.001	0.27
Attempts to reduce the amount of time online (0–4)	1.62 ± 1.35	2.21 ± 1.37	t = −4.86 (152)	*p* < 0.001	0.39
Problematic screen use	N = 59 (38.5%)	N = 93 (60.7%)	X^2^ = 23.13 (1)	*p* < 0.001	0.37

^1^ Paired *t*-test for ordinal variables, McNemar test for categorical variables. $: Effect size = Cohen’s d for all variables except “Problematic screen use”, for which Cramer’s V was calculated.

**Table 2:** Correlations between anxiety and current lifestyle habits (N = 153).

	Anxiety at T0		Anxiety at T1	
	r	*p* Value	r	*p* Value
Time spent with friends	−0.164	*p* = 0.043	−0.213	*p* = 0.008
Time spent watching TV	0.105	*p* = 0.154	0.044	*p* = 0.584
Time spent reading	0.168	*p* = 0.035	0.073	*p* = 0.369
Time spent inactive	0.235	*p* = 0.003	−0.037	*p* = 0.650
Time spent playing sports	−0.296	*p* = 0.001	−0.282	*p* < 0.001
Binge drinking	−0.076	*p* = 0.370	−0.150	*p* = 0.065
Binge eating	0.174	*p* = 0.046	0.362	*p* < 0.001
Cannabis use	0.064	*p* = 0.474	0.008	*p* = 0.926
Enough sleep	−0.072	*p* = 0.570	−0.283	*p* < 0.001
Early awakening	0.239	*p* = 0.003	0.191	*p* = 0.018
Difficulties in falling asleep	0.215	*p* = 0.015	0.332	*p* < 0.001
Daytime sleepiness	0.247	*p* = 0.002	0.383	*p* < 0.001
Time spent online	0.196	*p* = 0.013	0.070	*p* = 0.391
Staying up late to spend more time online	0.145	*p* = 0.061	0.181	*p* = 0.025
Little time to study because of time spent online	0.097	*p* = 0.197	0.196	*p* = 0.015
Attempts to reduce the amount of time spent online	0.217	*p* = 0.011	0.218	*p* = 0.007

**Table 3:** Variables associated with the change in anxiety level across time; multiple regression model (N = 153) *.

	*p*	Beta	Standardized *β*	Adjusted R2
Δ Difficulties in falling asleep	<0.001	+0.86	+0.27	
Δ Attempts to reduce screen time	0.005	+1.13	+0.21	0.199
Loneliness	0.011	−5.05	−0.19	
COVID-related experiences in the family	0.05			

* a positive value of Δ SCARED denotes an improvement in the SCARED total score across time, while a negative value of Δ SCARED denotes a worsening in the SCARED total score across time.

## Data Availability

The data presented in this study are openly available in Zenodo repository at DOI 10.5281/zenodo.10090891.
